# Efficient genome editing in a Mozambique tilapia cell line using CAS ribonucleoprotein complexes

**DOI:** 10.1038/s41598-026-42702-w

**Published:** 2026-03-26

**Authors:** Jiaqi Wang, Miroslav Bobrik, Nunticha Pankaew, Rémi Gratacap, Paul Digard, Tim P. Bean, Yehwa Jin, Diego Robledo

**Affiliations:** 1 The Roslin Institute and Royal (Dick) School of Veterinary Studies, The University of Edinburgh, Midlothian, UK; 2 Department of Pharmacy, UiT-The Arctic University of Norway, 9037 Tromsø, Norway; 3 Aquanzo Ltd, Roslin Innovation Centre, Easter Bush Campus, EH25 9RG Roslin, UK; 4 The Center for Aquaculture Technologies, San Diego, CA 92121 USA; 5 University of Santiago de Compostela, Santiago de Compostela, Spain

**Keywords:** Oreochromis mossambicus, aquaculture, CRISPR/Cas9, cell model, TiLV, Biological techniques, Biotechnology, Genetics, Molecular biology

## Abstract

Genome editing using the CRISPR/Cas system makes it possible to rapidly characterise gene function in vitro and in vivo, and provides a powerful platform through which the genetics of farmed fish can be altered to improve traits such as resistance to important pathogens. Tilapia is one of the most important farmed fish globally; however, its farming is heavily impacted by Tilapia lake virus (TiLV). The Mozambique tilapia (*Oreochromis mossambicus*) brain (OmB) cell line is susceptible to TiLV, making it an ideal in vitro model for studying host-pathogen interactions and mechanisms of disease resistance. To establish OmB cells as a model for gene editing in Tilapia, it is essential to optimise genome editing protocols. In this study, we optimized a CRISPR/Cas9-based genome editing system for OmB cells using ribonucleoprotein complexes. With the optimized protocol, we successfully edited two endogenous genes with efficiencies ranging from 67% to 70%. In conclusion, we establish a highly efficient CRISPR/Cas9 RNP based gene editing workflow optimized for OmB tilapia cell lines. This optimized platform will facilitate future functional genomic studies in tilapia and support the development of TiLV-resistant tilapia.

**Supplementary Information:**

The online version contains supplementary material available at 10.1038/s41598-026-42702-w.

## Introduction

Aquaculture is instrumental in supporting global food security, supplying vital high-quality protein to human diets. Fish currently account for about 17% of total global animal protein consumed, and in many low and middle income countries the share is over 50%^7^. Tilapia farming, a rapidly growing industry, is fundamental for the food security of the global South. Tilapia are among the five most produced groups of fish and are farmed in 127 countries around the world^3^. They are readily farmed because of their high proportion of edible meat, quick development and growth, tolerance to high-density culture, and resistance to common diseases^1^. Among cultured tilapia, Nile tilapia (*Oreochromis niloticus*) is the most important species and is the third most widely farmed finfish species globally in terms of production (4,514,615 t)^8^. In the current context of population growth and stagnation of fisheries, aquaculture production will need to double by 2050 to cope with the increased demand for aquatic goods^8^. Genetic improvement is expected to play a major role in this process, as faster growing, more resilient fish are fundamental for increasing the productivity of aquaculture while reducing its environmental footprint^14^. Selective breeding programmes, like the ongoing Genetically Improved Farmed Tilapia (GIFT) programme which selects for faster growth, will be fundamental in delivering constant improvement to traits of interest every generation. However, selective breeding has limitations, as (i) it depends on existing genetic variation in the population, (ii) provides relatively slow progress for traits with low heritability, and (iii) only a few traits can be selected each generation^18^. Conversely, genome editing can create novel genetic variation and can lead to large gains in traits of interest in a single generation, perhaps representing an ideal complement to current methods of selective breeding. Genome editing is especially interesting for disease resistance traits, as it may rapidly produce fully resistant animals. For example, researchers recently used CRISPR/Cas to edit the DNAJC14 gene in pig zygotes, generating healthy animals resistant to infection by two pestiviruses (classical swine fever virus (CSFV) and bovine viral diarrhoea virus (BVDV)^5^. In aquaculture species, genome editing has similarly been applied to investigate genetic resistance mechanisms, including CRISPR/Cas-mediated disruption of host genes influencing viral replication in fish cell lines and the identification of resistance associated loci in Atlantic salmon for infectious pancreatic necrosis virus^16,21,24,30^.

The rise of CRISPR/Cas technologies has facilitated the application of genome editing to several aquaculture species due to its efficiency, precision and cost-effectiveness^26^. Perhaps the main limitation in the application of genome editing is the identification of appropriate targets likely to lead to improvements in our traits of interest. Currently, gene function in aquaculture species is poorly understood, and mainly derived from homology with mammalian genes^25^. Cell lines represent ideal models to progress the functional characterization of fish genomes, and extensive progress has been made leveraging cell lines for the investigation of fish pathogens and the corresponding host responses^9^. Genome engineering of these cell lines represents a significant potential to propel advancements in the study of gene function, and for example, they have already been used in Atlantic salmon to study interferon signalling in viral responses^6^ and osmoregulatory mechanisms in Mozambique Tilapia (*O. mossambicus*) cells^15^. The process of gene editing is ultimately easier given integration of cas9 expression into the cell line genome, but this is not always feasible. Cas9 is often delivered in alternative forms, such as purified protein, plasmid or mRNA. Due to the limited amount of functional information available on fish cell lines, using RNP directly for gene editing is beneficial; it does not require promoter optimization and given efficient entry into the cell is more likely to function as expected. In comparison to lentivirus infections and plasmid delivery, it has been demonstrated that the RNP complex delivery avoids the prolonged expression and potential integration associated with viral or plasmid vectors.

In this study we present a straightforward and replicable protocol for editing the Mozambique tilapia brain (OmB) cell line with high efficiency via the electroporation of Cas9 RNP complexes. This optimized protocol provides a more convenient platform for the investigation of gene function in tilapia, a necessary step prior to the application of genome editing for genetic improvement in vivo. In particular, the OmB cell line represents a valuable model to study host-pathogen interactions, including with Tilapia Lake Virus (TiLV) infection, and therefore to discover candidate resistance genes.

## Materials and methods

### Cell Culture

OmB cells (ATCC CRL-3481), a fibroblast-like cell line derived from the brain of a Mozambique tilapia, were cultured in L15 media (Sigma-Aldrich, St. Louis, USA), supplemented with 10% (v/v) fetal bovine serum (FBS, Gibco), 2 mM glutamine, 100 µg/mL penicillin, and 100 U/mL streptomycin (Pen/Strep, Gibco) at 28 °C. When cells reached 80–90% confluency, cells were washed with phosphate-buffered saline (PBS), trypsinized (Trypsin-EDTA (0.25%) solution, Gibco), harvested by mixing with equal volume of the complete growth media, and centrifuged (400 g, 4 min). Then, cells were passaged at a 1:4 ratio with fresh media. Cell passage number was monitored across experiments, and all assays were performed using cells within a comparable passage range to minimize culture associated variability.

### Initial optimisation of electroporation setting using tracrRNA-ATTO550

OmB cells were harvested, resuspended in Opti-MEM and the number of cells counted with a Hemocytometer. Cells were centrifuged again and resuspended in Opti-MEM medium at a concentration of 10^7^ cells/mL. The ATTO550-tracrRNA (IDT) was resuspended in nuclease-free water and added to 10^5^ cells in different concentrations (1 µM, 2 µM, 3 µM). The 10 µL mix of cells and tracrRNA-ATTO550 was electroporated in a Neon transfection system (Invitrogen, Carlsbad, USA) using three different electroporation settings (1700v 15ms 2pulses, 1400v 20ms 2pulses, 1400v 20ms 1pulses). Twenty-four hours post electroporation, cells positive for ATTO550 were quantified by flow cytometry (LSRFortessa, BD Biosciences) to provide an estimate of the transfection rate of each setting. As negative controls, cells were not incubated with ATTO550, or were incubated with different concentrations of ATTO550 cells without electroporation. The fluorescence level of these negative controls was set as baseline to detect ATTO550-positive cells. The optimal result from tracrRNA-ATTO550 electroporation was used as starting point to define and test electroporation settings for editing efficiency and cell survival in a GFP-expressing OmB cell line.

### Optimisation of electroporation settings using Cas9 RNP

GFP was targeted for editing with the GFP guide RNA sequence “GAGCTGGACGGCGACGTAAA”^29^. The RNP was made by mixing an equimolar mixture of eGFP crRNA (1.4 µL, 200 µM), tracrRNA (1.4 µL, 200 µM), and Nuclease-Free Duplex Buffer (2.2 µL), incubating at 95 °C for 5 min and then cooling to room temperature (RT) to form 56 µM duplex gRNA. The crRNA: tracrRNA duplex (0.5 µL, 28 pmol) was then combined with 0.38µL (23.6 pmol) Alt-R^®^ *Streptococcus pyogenes* Cas9 Nuclease V3 (62 µM, IDT, Cat. No. 1081058) mixed with 0.12 µL Opti-MEM medium (Gibco), followed by a 20-minute incubation at room temperature to form 23.6 µM Ribonucleoprotein (RNP) complexes.

OmB-GFP cells are OmB cells that have randomly integrated the pEGFP-puro plasmid (17,448, Addgene) into their genome. In this plasmid, GFP expression is driven by the strong CMV promoter, allowing the cells to show stable and continuous green fluorescence. After transfection, puromycin selection was applied, and only the cells that successfully integrated the plasmid survived. As a result, the final cell population maintains stable GFP expression. OmB-GFP cells to be used for optimising electroporation were washed with PBS, trypsinized, centrifuged (500x*g*, 4 min), and then resuspended in Opti-MEM medium. The number of cells was then counted using a hemocytometer. Cells were centrifuged again and resuspended in Opti-MEM medium to a concentration of 10^7^ cells/mL. A total of 10^5^ cells in ten µL were mixed with 1 µL of 2.36 µM RNP and 1 µL of Opti-MEM for a final RNP concentration of 2 µM in a volume of 10 µL. After incubation at room temperature for 5 minutes, the mixture was electroporated using wider electroporation conditions (voltage 1400 to 1800 V, pulse duration 10 to 30 ms, number of pulses 1 to 3) with the Neon system (Invitrogen, Carlsbad, USA) according to the manufacturer’s instructions, to test the best editing settings, but follow the published protocol using OptiMEM instead of Neon Buffers^10^. Opti-MEM was used in place of the proprietary Neon buffer to ensure compatibility with ribonucleoprotein complexes, reduce buffer related variability across experiments, and maintain consistency with previously validated RNP-based electroporation protocols in fish cell lines. The GFP-RNP was electroporated using 10 µL Neon tips and dispensed into 1 mL of OmB normal cell culture media in a 24-well plate. These cells were used to test editing efficiency, cell survival and loss of EGFP as described below.

### Editing efficiency

Genomic DNA from edited cells was amplified by PCR and Sanger sequenced to assess editing efficiency. Genomic DNA was extracted from edited cells using QuickExtract buffer (Lucigen, Middleton, USA) according to the manufacturer’s instructions. The target genomic region was amplified by a single round of PCR using NEB Q5 polymerase in 50 µL reaction volume. One microliter of extracted genomic DNA was used as template per reaction, and PCR was performed for 33 cycles with an annealing temperature of 65 °C. PCR products were verified by agarose gel electrophoresis, purified, and directly subjected to Sanger sequencing (GATC/Eurofins). The resulting chromatograms were analyzed using ICE and TIDE to quantify editing efficiency and indel profiles. Both ICE and TIDE were used for initial analysis, yielding comparable editing efficiency estimates. For consistency, ICE outputs were used for all quantitative values reported in the results.

### Cell survival

Cell viability was assessed using CellTiter-Glo 2.0 (Invitrogen). The CellTiter-Glo^®^ 2.0 Assay use luminescence to measure adenosine triphosphate (ATP), an indicator of metabolically active cells; the level of luminescence is directly correlated with the population of viable cells within the culture. Cells incubated with Cas9 RNP complexes but not subjected to electroporation were included as controls. After electroporation, 100 µL of electroporated cells were transferred to a 96-well plate and incubated for 48 h. Cells were rinsed once with 200 µL of PBS, retaining only surviving cells adherent to the bottom of the plate. Subsequently, 120 µL of CellTiter-Glo solution, diluted 1:10 in PBS, was added to each well. The plate was then incubated in darkness for 30 min on a plate rocker at room temperature. Next, 100 µL of the resulting solution from each well was carefully transferred to a flat-bottom white 96-well plate (Greiner Bio-One, Austria). The luminescence emitted was measured using a Cytation3 imaging reader, and the Gen5 software V3.03 (BioTek, Winooski, USA) was employed for data analysis. Non-electroporated OmB-GFP cells were used as control.

### Loss of eGFP signal

Loss of GFP expression in the edited OmB-GFP cells was quantified by flow cytometry 14 days after electroporation, using unedited OmB-GFP cells as a control. Cells were detached using trypsinization and suspended in phosphate-buffered saline (PBS). Maintaining the cells on ice, flow cytometry analysis was performed employing a LSRFortessa X-20 (BD Biosciences, San Jose, USA). During data acquisition, single-cell events were identified and gated to ensure accurate analysis. The percentage of GFP-positive cells and the intensity of GFP fluorescence from each individual cell were quantitatively measured.

### Editing efficiency in endogenous genes

After optimizing the electroporation and incubation conditions for the OmB cell line as described above, we implemented the final optimised protocol to target the endogenous genes *slc45a2* (solute carrier family 45 member 2) and *otud4* (OTU Deubiquitinase 4) (The genes *slc45a2* and *otud4* were selected as representative targets because they are non-essential for cell viability and have well-characterized genomic sequences, making them suitable markers for assessing editing efficiency.). The sequences of both genes were extracted from the Nile tilapia (*O. niloticus*) NCBI genome assembly GCF_001858045.2. *Slc45a2* and *otud4* gene sequences were compared with an unpublished Mozambique tilapia genome assembly (reads available on the European Nucleotide Archive, ERR8978383), shared by Dr Wilfried Haerty (Earlham Institute). All crRNAs were designed using CRISPOR (http://crispor.tefor.net/) and the CRISPR Design Tool from Synthego Inc. The crRNA design prioritized sequences targeting the first half of the coding sequence with high on-target scores, targeting sequences conserved across Nile and Mozambique tilapia genomes. Factors considered included potential off-targets in the tilapia genome and maintaining a GC content between 40% and 70%. Self-complementarity between the selected crRNA sequence and the tracrRNA was also considered as it can hinder editing efficiency. The chosen crRNA sequences for *slc45a2* and *otud4* (Table 1) were obtained from IDT in the proprietary Alt-R format (IDT, Coralville, IA, USA). Following electroporation, the cells were cultured for one week before undergoing Sanger sequencing to evaluate genome editing efficiency (primers shown in Table 1). All experiments were performed using two or three independent biological replicates. Experiments involving optimization of electroporation parameters and GFP editing efficiency were conducted with two biological replicates, consistent with their technical and exploratory nature.

**Table 1:** Endogenous gene crRNA sequences and primers used for amplification and sequencing of target regions.

Target	Guide sequences (5’-3)’	Primers (5’-3)’	Annealing temperature (°C)
*otud4*	GATGACTACTTGAAATCGAT	F: GGAAAACGAAACTGGACGCT R: TTTGCTCTCCCACGATGTTGT	67
*slc45a2*	TAAGGCCTCCGCCGGCCCCA	F: CAGCACTTCTCTGCACCAGTA R: CCTGAATCCAAACTGGGAGCC	68

## Results

### Initial optimisation of electroporation settings using tracrRNA-ATTO550 in OmB cells

**Fig. 1:**
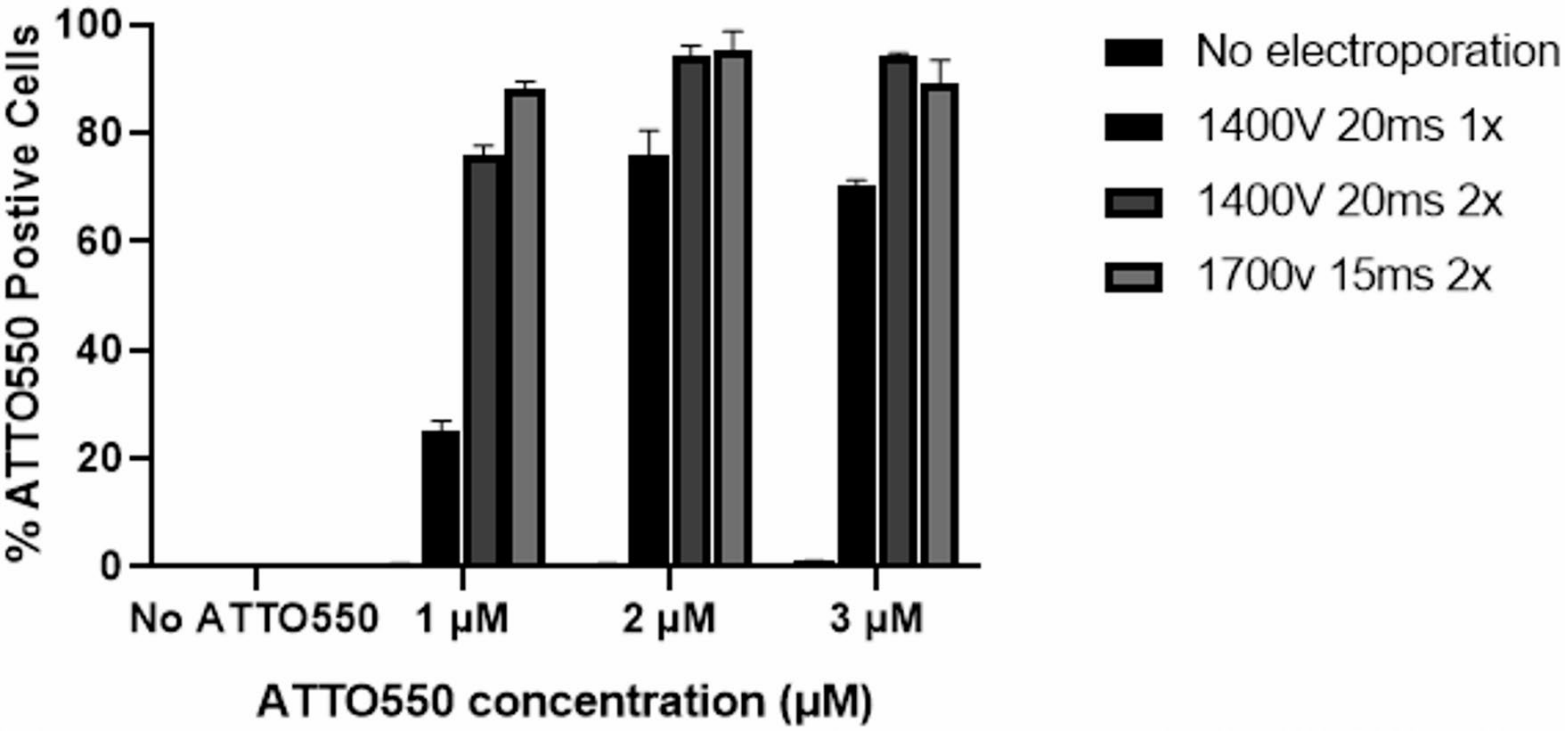
Initial optimisation of electroporation setting with tracrRNA-ATTO550. Different tracrRNA-ATTO550 concentrations (1, 2 and 3 µM) were electroporated using three different electroporation conditions. Mock controls were not incubated with tracrRNA-ATTO550 but electroporated, while No electroporation controls were incubated with tracrRNA-ATTO550 but not-electroporated. The percentage of ATTO550 positive cells was measured using flow cytometry 24 h post electroporation. The error bars represent standard deviation (*n* = 3 biological replicates).

TracrRNA-ATTO550 was used to determine the minimum RNP concentration required for efficient transfection in OmB cells. Red fluorescence, detected by flow cytometry, served as a readout for electroporation efficiency. Three different tracrRNA-ATTO550 concentrations (1, 2 and 3 µM) were tested under three different electroporation conditions:1400 V 20ms 1 pulse, 1400 V 20 ms 2 pulses, and 1700 V 15ms 2 pulses, along with two negative controls: cells with no electroporation and cells without tracrRNA-ATTO550. All conditions were tested in three independent experiments. Across the conditions tested, transfection efficiency at 1 µM was generally lower than at higher RNP concentrations, although differences were modest under some electroporation settings. In contrast, 2 and 3 µM achieved similar efficiencies, reaching nearly 100% transfection efficiency in two of the electroporation conditions tested (Fig. 1). Since transfection efficiency plateaued at 2 µM, this concentration was chosen for subsequent experiments as it would minimise potential toxicity, reduce off-target effects, and provide a more economical approach.

### Optimising electroporation by targeting gfp using Cas9 RNP in OmB-GFP cells

**Fig. 2:**
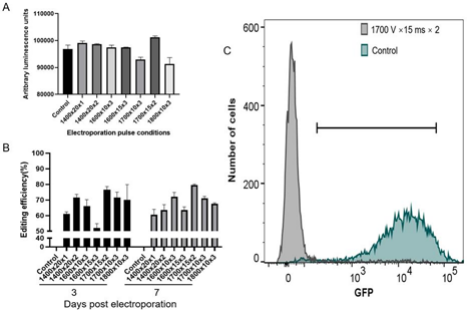
Efficient knockout of GFP in OmB-GFP cells. (**A**) Cell survival of OmB-GFP cells was assessed for different electroporation parameters at 24 h post electroporation, (**B**) Editing efficiency (GFP) was evaluated using Sanger sequencing and ICE analysis at both 3 and 7 days post electroporation, (**C**) GFP fluorescence levels 14 days after electroporation, quantified using flow cytometry. Error bars in Fig. 2B and C represent standard deviation (*n* = 2 independent biological replicates).

To establish the optimal conditions for gene editing, eGFP-cRNA was used to knock out EGFP expression in the OmB-GFP cell line. Multiple electroporation parameters were tested, including voltages of 1400, 1600, 1700–1800 V, pulse durations of 10, 15 or 20ms, and pulse numbers of 1, 2 or 3 pulses, in two independent experiments. Cell survival 48 h post-electroporation was assessed using CellTiter-Glo and tested in two independent experiments (Fig. 2A). Luminescence measurements indicated minor differences in cell viability across conditions, with survival ranging from 95 to 105%, demonstrating that all tested settings were well tolerated. Genome editing efficiency was evaluated by Sanger sequencing of PCR fragments from the GFP locus (Fig. 2B). The condition 1700 V 15ms 2 pulses yielded the highest editing efficiency at both 3 and 7 days post-electroporation, reaching nearly 80%. This optimal parameter set was further validated by flow cytometry, measuring the number of cells showing GFP fluorescence (Fig. 2C). In the control group, 90.3% of cells were GFP-positive, compared to only 6.62% in the edited group, confirming high knockout efficiency under these conditions.

### Editing efficiency in endogenous tilapia genes

**Fig. 3:**
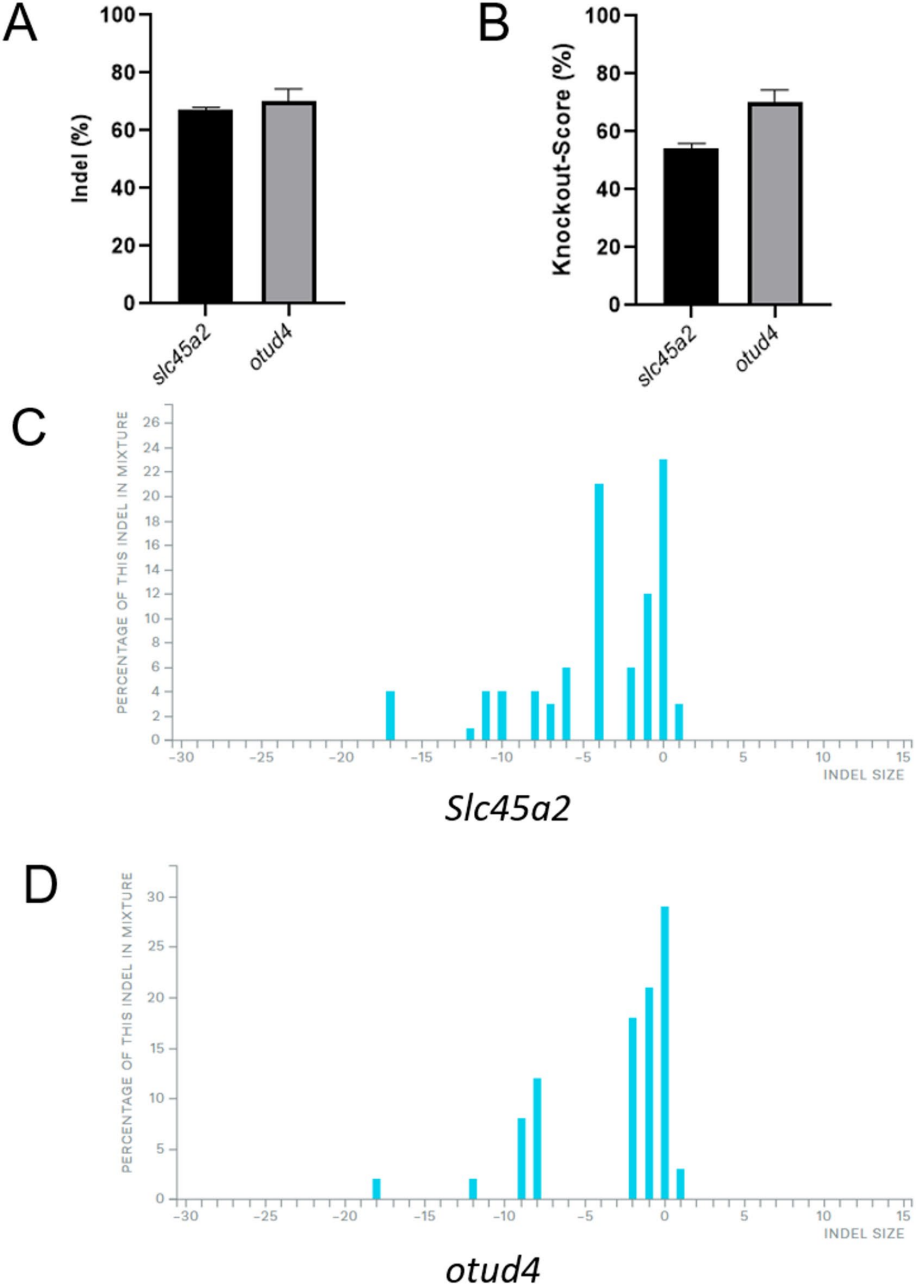
Editing efficiency for endogenous tilapia genes. Editing efficiency and knock-out efficiency was measured using Sanger sequencing and ICE analysis (**A**, **B**). The error bars represent standard deviation (*n* = 3 biological replicates). The indel plot visually represents the size of insertions or deletions in a DNA sequence, indicating the number of added (+) or removed (–) nucleotides for slc45a2 (**C**), otud4 (**D**). These plots also illustrates the percentage of genomes that exhibit each specific insertion or deletion.

Finally, to assess the potential of CRISPR/Cas9-mediated gene editing in the OmB cell line, we targeted the endogenous genes *slc45a2* and *otud4* using the previously optimised conditions (2 µM RNP and 1700 v x 15 ms x 2 pulses). Both genes demonstrated a high editing efficiency of 67% (*slc45a2*) and 70% (*otud4*) (Fig. 3A), the corresponding knock-out scores were 54.3%(*slc45a2*) and 62% (*otud4*) (Fig. 3B). Sanger sequencing of PCR products from the target loci revealed multiple base deletions and insertions (Fig. 3C, D), indicating successful editing of both genes. All Sanger sequencing traces are provided in addition file Fig. S1. These results demonstrate that the OmB cell line can be efficiently edited at multiple endogenous loci, supporting its technical use as a platform for genome editing applications in tilapia.

## Discussion

In this study, we optimized a method of RNP delivery for gene editing of the Mozambique tilapia cell line, OmB. In comparison to previous studies utilizing Invitrogen Lipofectamine CRISPRMAX transfection reagent system our process resulted in significantly higher transfection efficiencies^13^. In addition, gene editing with Lipofectamine was unsuccessful, further highlighting the effectiveness of this optimized electroporation approach for efficient delivery and successful gene editing in OmB cells^13^. We confirmed the optimal concentration of tracrRNA reagents (RNP concentration) by conjugating with the ATTO550 to create a simple reporter in electroporation methods. When using 2 µM RNP we achieved a transfection rate approaching 100% across a range of electroporation settings. This was similar to results previously reported in four medaka cell lines where electroporation using ATTO550 resulted in transfection efficiency exceeding 90%^20^.

Following confirmation of the optimal RNP concentrations, we built on these results by testing multiple electroporation variables such as voltage (1400–1800 V), time (10–20 ms), and pulse number (1–3). To characterise this process, we utilised the OmB-GFP expressing cell line, and knocked-out GFP using a previously reported guide RNA^29^. By using these settings, it was also possible to remove virtually all GFP fluorescence suggesting successful functional knockout of this protein. The differences in optimal electroporation settings compared to previous studies using plasmids may be attributed to variations in cargo or the use of OptiMEM instead of buffer R for electroporation^15,28^. Increasing the duration and number of pulses enhances the mutation rate of electroporation-mediated genome editing. This is associated with an increase in pore density and size, allowing more genome editing components to enter the cells. However, escalating parameters to improve transfection efficiency can have adverse effects on cell survival, further emphasizing the need to strike a balance between editing efficiency and cell survival when optimizing electroporation parameters^19^. Electroporated cells exhibit membrane damage, increased intracellular Ca^2+^ concentration, mitochondrial disruption, ATP depletion, ROS generation, and DNA damage, all of which can lead to various forms of cell death^2^. Our cells demonstrated both high survival and high editing efficiency suggesting an ideal balance between cellular damage and cargo delivery.

Successful editing of the endogenous genes *slc45a2* and *otud4* in the OmB cell line further confirmed the practical applicability of our optimized protocol. The gene editing efficiency is higher than that of the currently reported in Medaka fish cells (average 50% from tide analysis)^20^, where an RNP-based delivery method was also employed for cell line gene editing. These efficiency values are well within expected successful range and may even be further improved in future by optimising guide RNA sequences for target genes. The first application of the CRISPR/Cas9 system to suppress viral infections in aquatic animal cell lines was reported in 2018^21^. Since then, its use has rapidly advanced in cyprinid and salmonid cell lines^4,16,17,24,30^. Our establishment of a gene editing method in the OmB cell line provides a foundation for future investigations of genetic resistance to infectious diseases in cichlid species.

Compared to alternative gene editing approaches in OmB cell lines, RNP delivery offers a notable advantage by circumventing the necessity for endogenous promoter utilization to achieve high gene editing efficiencies, thus presenting fewer constraints. Current methodologies for gene editing in tilapia cell lines predominantly rely on the integration of Cas9 into the OmB cell line^13,15^. However, this approach raises concerns regarding potential off-target effects stemming from the constitutive expression of Cas9, as well as uncertainties surrounding its long-term cellular impact. Editing efficiencies reported here are lower than those achieved in some salmonid cell lines using RNP-based approaches (e.g. ~90%^10^). Fish cell lines differ substantially in their cellular origin, membrane composition, genome organization, and optimal culture conditions, all of which can influence electroporation efficiency and genome editing outcomes. As such, editing efficiency benchmarks established in salmonid systems may not be directly transferable to cichlid derived cell lines such as OmB. Nevertheless, in contrast to salmonid cell lines, OmB cells are also readily cloneable from single cells, which in principle facilitates the future derivation of stable homozygous knockout lines when combined with high editing efficiency, although this was not directly assessed in the present study.

A key advantage of Sanger deconvolutional analyses such as ICE is their ability to estimate genome editing efficiency using Sanger traces, eliminating the need for deep sequencing or individual mutant cloning. We did not evaluate off-target effects in this study, as these are largely set by guide RNA design; users should assess potential off-target sites for their specific guides. A limitation of the present study is that genome editing outcomes were assessed exclusively at the DNA level using Sanger sequencing deconvolution approaches (ICE and TIDE). The loci were selected as representative endogenous targets to benchmark editing efficiency rather than as functional targets. Future studies incorporating transcriptomic, proteomic, or phenotypic analyses will be required to link efficient genome editing with functional outcomes.

## Conclusions

We report here an optimised gene editing method based on the CRISPR/Cas9 system in the Mozambique tilapia brain cell line (OmB) by delivering RNP complexes consisting of Cas9 and crRNA: tracrRNA duplexes through electroporation. Through optimization, we determined that the optimal concentration of RNP is 2 µM, and the best electroporation settings are 1700 V 15ms 2 pulses. When testing these parameters against endogenous genes, we achieved over 60% knockout efficiency. This optimization scheme will facilitate functional genetic studies in the Mozambique tilapia brain cell line.

## Supplementary Information

Below is the link to the electronic supplementary material.

Supplementary Material 1

## Data Availability

The datasets used and/or analysed during the current study, Sanger sequencing was performed in this study for validation purposes. The validated Sanger sequences are provided in the Supplementary Information, and the raw trace files (.ab1) are available from the corresponding author upon reasonable request.

## References

[1] Abwao, J., Jung’a, J., Barasa, J.E., Kyule, D., Opiyo, M., Awuor, J.F., Ogello, E., Munguti, J.M. & Keya, G.A. Selective breeding of Nile tilapia, Oreochromis niloticus: A strategy for increased genetic diversity and sustainable development of aquaculture in Kenya. *J. Appl. Aquac*. **35**, 237–256. 10.1080/10454438.2021.1958728 (2023).

[2] Batista Napotnik, T., Polajžer, T. & Miklavčič, D. Cell death due to electroporation—A review. *Bioelectrochem. Amst Neth.* **141**, 107871. 10.1016/j.bioelechem.2021.107871 (2021). 10.1016/j.bioelechem.2021.107871 34147013

[3] Cai, J. Top 10 species groups in global aquaculture 2017. Rome, Italy: FAO. (2019). https://www.fao.org/documents/card/en/c/ca5224en/ (Accessed July 4, 2023).

[4] Collins, C., Chaumont, L., Peruzzi, M., Jamak, N., Boudinot, P., Béjar, J., Moreno, P., Álvarez Torres, D. & Collet, B. Effect of a loss of the mda5/ifih1 gene on the antiviral resistance in a Chinook salmon Oncorhynchus tshawytscha cell line. *PLoS ONE*. **19**10.1371/journal.pone.0311283 (2024). 10.1371/journal.pone.0311283 PMC11472919 39401233

[5] Crooke, H., Schwindt, S., Fletcher, S.L., Isken, O., Harding, S., Berkley, N., Tait-Burkard, C., Warren, C., Whitelaw, C.B.A., Tautz, N. & Lillico, S. GDNAJC14 gene-edited pigs are resistant to classical pestiviruses. *Trends Biotechnol. press.*10.1016/j.tibtech.2025.09.008 (2025). 10.1016/j.tibtech.2025.09.008 41130838

[6] Dehler, C.E., Lester, K., Della Pelle, G., Jouneau, L., Houel, A., Collins, C., Dovgan, T., Machat, R., Zou, J., Boudinot, P. & Martin, S.A. Viral Resistance and IFN signaling in STAT2 knockout fish cells. *J. Immunol. Baltim. Md. 1950*. **203**, 465–475. 10.4049/jimmunol.1801376 (2019). 10.4049/jimmunol.1801376 PMC6612602 31142600

[7] FAO. *The State of World Fisheries and Aquaculture 2020: Sustainability in action* (FAO, 2020). 10.4060/ca9229en

[8] FAO. FishStatJ: Universal software for fishery statistical time series: aquaculture production. (2022).

[9] Goswami, M., Yashwanth, B. S., Trudeau, V. & Lakra, W. S. Role and relevance of fish cell lines in advanced in vitro research. *Mol. Biol. Rep.* **49**, 2393–2411. 10.1007/s11033-021-06997-4 (2022). 35013860 10.1007/s11033-021-06997-4 PMC8747882

[10] Gratacap, R. L., Jin, Y. H., Mantsopoulou, M. & Houston, R. D. Efficient genome editing in multiple salmonid cell lines using ribonucleoprotein complexes. *Mar. Biotechnol.* **22**, 717–724. 10.1007/s10126-020-09995-y (2020). 10.1007/s10126-020-09995-y PMC7520412 32946000

[11] Hamar, J., Cnaani, A. & Kültz, D. Effects of CRISPR/Cas9 targeting of the myo-inositol biosynthesis pathway on hyper-osmotic tolerance of tilapia cells. *Genomics* **116**10.1016/j.ygeno.2024.110833 (2024a). 10.1016/j.ygeno.2024.110833 38518899

[12] Hamar, J., Cnaani, A. & Kültz, D. Transcriptional upregulation of the myo-inositol biosynthesis pathway is enhanced by NFAT5 in hyperosmotically stressed tilapia cells. *Am. J. Physiol. Cell. Physiol.* **327**, C545–C556. 10.1152/ajpcell.00187.2024 (2024b). 38946247 10.1152/ajpcell.00187.2024

[13] Hamar, J. & Kültz, D. An efficient vector-based CRISPR/Cas9 system in an Oreochromis mossambicus cell line using endogenous promoters. *Sci. Rep.* **11**, 7854. 10.1038/s41598-021-87068-3 (2021). 33846462 10.1038/s41598-021-87068-3 PMC8041756

[14] Houston, R.D., Bean, T.P., Macqueen, D.J., Gundappa, M.K., Jin, Y.H., Jenkins, T.L., Selly, S.L.C., Martin, S.A., Stevens, J.R., Santos, E.M., Davie, A & Diego, R. Harnessing genomics to fast-track genetic improvement in aquaculture. *Nat. Rev. Genet.* **21**, 389–409. 10.1038/s41576-020-0227-y (2020). 32300217 10.1038/s41576-020-0227-y

[15] Kim, C., Cnaani, A. & Kültz, D. Removal of evolutionarily conserved functional MYC domains in a tilapia cell line using a vector-based CRISPR/Cas9 system. *Sci. Rep.* **13**, 12086. 10.1038/s41598-023-37928-x (2023). 37495710 10.1038/s41598-023-37928-x PMC10371998

[16] Kim, M. S., Shin, M. J. & Kim, K. H. Increase of viral hemorrhagic septicemia virus growth by knockout of IRF9 gene in Epithelioma papulosum cyprini cells. *Fish. Shellfish Immunol.* **83**, 443–448. 10.1016/j.fsi.2018.09.025 (2018). 30244086 10.1016/j.fsi.2018.09.025

[17] Kwak, J. S. & Kim, K. H. Generation of self-inhibitory recombinant viral hemorrhagic septicemia virus (VHSV) by insertion of viral P gene-targeting artificial MicroRNA into viral genome and effect of dicer gene knockout on the recombinant VHSV replication. *Mar. Biotechnol.* **23**, 546–559. 10.1007/s10126-021-10045-4 (2021). 10.1007/s10126-021-10045-4 34268626

[18] Labroo, M. R., Studer, A. J. & Rutkoski, J. E. Heterosis and hybrid crop breeding: A multidisciplinary review. *Front. Genet.* **12**. (2021). 10.3389/fgene.2021.643761 10.3389/fgene.2021.643761 PMC7943638 33719351

[19] Lin, J. C. & Van Eenennaam, A. L. Electroporation-mediated genome editing of livestock zygotes. *Front. Genet.* **12**, 648482. 10.3389/fgene.2021.648482 (2021). 33927751 10.3389/fgene.2021.648482 PMC8078910

[20] Liu, Q., Yuan, Y., Zhu, F., Hong, Y. & Ge, R. Efficient genome editing using CRISPR/Cas9 ribonucleoprotein approach in cultured Medaka fish cells. *Biol. Open.* **7**, bio035170. 10.1242/bio.035170 (2018). 30072445 10.1242/bio.035170 PMC6124564

[21] Ma, J., Fan, Y., Zhou, Y., Liu, W., Jiang, N., Zhang, J. & Zeng, L. Efficient resistance to grass carp reovirus infection in JAM-A knockout cells using CRISPR/Cas9. *Fish. Shellfish Immunol.* **76**, 206–215. 10.1016/j.fsi.2018.02.039 (2018). 29477498 10.1016/j.fsi.2018.02.039

[22] Martínez-López, A., Chinchilla, B., Encinas, P., Gomez-Casado, E., Estepa, A. & Coll, J.M. Replacement of the human cytomegalovirus promoter with fish enhancer and core elements to control the expression of the G gene of viral haemorrhagic septicemia virus (VHSV). *J. Biotechnol.* **164**, 171–178. 10.1016/j.jbiotec.2012.08.005 (2012). 22954890 10.1016/j.jbiotec.2012.08.005

[23] Naylor, R.L., Hardy, R.W., Buschmann, A.H., Bush, S.R., Cao, L., Klinger, D.H., Little, D.C., Lubchenco, J., Shumway, S.E. & Troell, M.A. 20-year retrospective review of global aquaculture. *Nature* **591**, 551–563. 10.1038/s41586-021-03308-6 (2021). 33762770 10.1038/s41586-021-03308-6

[24] Pavelin, J., Jin, Y.H., Gratacap, R.L., Taggart, J.B., Hamilton, A., Verner-Jeffreys, D.W., Paley, R.K., Rubin, C.J., Bishop, S.C., Bron, J.E., Robledo, D & Houston, R.D. The nedd-8 activating enzyme gene underlies genetic resistance to infectious pancreatic necrosis virus in Atlantic salmon. *Genomics* **113**, 3842–3850. 10.1016/j.ygeno.2021.09.012 (2021). 34547402 10.1016/j.ygeno.2021.09.012 PMC8682971

[25] Rasal, K. D., Iquebal, M. A., Dixit, S., Vasam, M., Raza, M., Sahoo, L., Jaiswal, S., Nandi, S., Mahapatra, K. D., Rasal, A., Udit, U. K., Meher, P. K., Murmu, K., Angadi, U., Rai, A., Kumar, D., & Sundaray, J. K. Revealing Alteration in the Hepatic Glucose Metabolism of Genetically Improved Carp, Jayanti Rohu Labeo rohita Fed a High Carbohydrate Diet Using Transcriptome Sequencing. *Int. J. Mol. Sci.* **21**, 8180. 10.3390/ijms21218180 (2020). 33142948 10.3390/ijms21218180 PMC7662834

[26] Robinson, N.A., Østbye, T.K.K., Kettunen, A.H., Coates, A., Barrett, L.T., Robledo, D. & Dempster, T. A guide to assess the use of gene editing in aquaculture. *Rev. Aquac*. **16**, 775–784. 10.1111/raq.12866 (2024).

[27] Schiøtz, B.L., Rosado, E.G., Baekkevold, E.S., Lukacs, M., Mjaaland, S., Sindre, H., Grimholt, U. & Gjøen, T. Enhanced transfection of cell lines from Atlantic salmon through nucoleofection and antibiotic selection. *BMC Res. Notes*. **4**, 136. 10.1186/1756-0500-4-136 (2011). 21548922 10.1186/1756-0500-4-136 PMC3113957

[28] Segev-Hadar, A., Slosman, T., Rozen, A., Sherman, A., Cnaani, A. & Biran, J. Genome editing using the CRISPR-Cas9 system to generate a solid-red germline of nile tilapia (*Oreochromis niloticus*). *CRISPR J.* **4**, 583–594. 10.1089/crispr.2020.0115 (2021). 34406049 10.1089/crispr.2020.0115

[29] Shalem, O., Sanjana, N.E., Hartenian, E., Shi, X., Scott, D.A., Mikkelsen, T.S., Heckl, D., Ebert, B.L., Root, D.E., Doench, J.G. & Zhang, F. Genome-scale CRISPR-Cas9 knockout screening in human cells. *Science* **343**, 84–87. 10.1126/science.1247005 (2014). 24336571 10.1126/science.1247005 PMC4089965

[30] Xu, C., Gamil, A. A. A., Wang, X., Munang’andu, H. M. & Evensen, Ø. MAVS disruption impairs downstream signaling and results in higher virus replication levels of salmonid alphavirus subtype 3 but not infectious pancreatic necrosis virus in vitro. *Front. Immunol.* **15**10.3389/fimmu.2024.1401086 (2024). 10.3389/fimmu.2024.1401086 PMC11187282 38903507

